# The Resistance of Cancer Cells to Palbociclib, a Cyclin-Dependent Kinase 4/6 Inhibitor, is Mediated by the ABCB1 Transporter

**DOI:** 10.3389/fphar.2022.861642

**Published:** 2022-03-08

**Authors:** Han Fu, Zhuo-Xun Wu, Zi-Ning Lei, Qiu-Xu Teng, Yuqi Yang, Charles R. Ashby, Yixiong Lei, Yuyin Lian, Zhe-Sheng Chen

**Affiliations:** ^1^ School of Public Health, Guangzhou Medical University, Guangzhou, China; ^2^ Department of Pharmaceutical Sciences, College of Pharmacy and Health Sciences, St. John’s University, Queens, NY, United States; ^3^ Guangdong Provincial Key Laboratory of Digestive Cancer Research, Precision Medicine Center, The Seventh Affiliated Hospital, Sun Yat-Sen University, Shenzhen, China

**Keywords:** ATP-binding cassette transporter, ABCB1, palbociclib, multidrug resistance, CDK4/6 inhibitor

## Abstract

Palbociclib was approved by the United States Food and Drug Administration for use, in combination with letrozole, as a first-line treatment for estrogen receptor-positive/human epidermal growth factor receptor 2-negative (ER+/HER2-) postmenopausal metastatic breast cancer. However, recent studies show that palbociclib may be an inhibitor of the ABCB1 transporter, although this remains to be elucidated. Therefore, we conducted experiments to determine the interaction of palbociclib with the ABCB1 transporter. Our *in vitro* results indicated that the efficacy of palbociclib was significantly decreased in the ABCB1-overexpressing cell lines. Furthermore, the resistance of ABCB1-overexpressing cells to palbociclib was reversed by 3 μM of the ABCB1 inhibitor, verapamil. Moreover, the incubation of ABCB1-overexpressing KB-C2 and SW620/Ad300 cells with up to 5 μM of palbociclib for 72 h, significantly upregulated the protein expression of ABCB1. The incubation with 3 µM of palbociclib for 2h significantly increased the intracellular accumulation of [^3^H]-paclitaxel, a substrate of ABCB1, in ABCB1 overexpressing KB-C2 cells but not in the corresponding non-resistant parental KB-3-1 cell line. However, the incubation of KB-C2 cells with 3 μM of palbociclib for 72 h decreased the intracellular accumulation of [^3^H]-paclitaxel due to an increase in the expression of the ABCB1 protein. Palbociclib produced a concentration-dependent increase in the basal ATPase activity of the ABCB1 transporter (EC_50_ = 4.73 μM). Molecular docking data indicated that palbociclib had a high binding affinity for the ABCB1 transporter at the substrate binding site, suggesting that palbociclib may compete with other ABCB1 substrates for the substrate binding site of the ABCB1. Overall, our results indicate that palbociclib is a substrate for the ABCB1 transporter and that its *in vitro* anticancer efficacy is significantly decreased in cancer cells overexpressing the ABCB1.

## Introduction

Multidrug resistance (MDR) in tumors is defined as the development of resistance to structurally and mechanistically unrelated classes of anticancer drugs (Szakács et al., 2006). MDR is a major cause of cancer chemotherapy failure, which can lead to tumor recurrence and the death of cancer patients (Lage and Dietel, 2000). MDR can occur due to 1) the overexpression of certain adenosine triphosphate (ATP)-binding cassette (ABC) transporter proteins (Robey et al., 2018); 2) an increase in the repair of damaged DNA (Helleday et al., 2008); 3) mutations in the target of the anticancer drugs which decrease or abrogate the efficacy of these drugs (Chandrasekhar et al., 2019); 4) an increased tolerance to the stressful tumor microenvironment (TME) (Erin et al., 2020; Seebacher et al., 2021); 5) evasion of programmed cell death (Shahar and Larisch, 2020; Neophytou et al., 2021); 6) higher levels of reactive oxidative species (Cui et al., 2018); 7) an increase in the biotransformation of the anticancer drugs to less active or inactive metabolites (Zaal and Berkers, 2018); 8) sequestration of drugs by organelles or intracellular molecules that decrease their interaction with their cellular target(s), and 9) specific long noncoding RNAs (lncRNAs) (Liu et al., 2020). Numerous studies have shown that one of the primary mechanisms that mediates MDR in cancer cells is the overexpression of ABCB1 (i.e., P-gp or MDR1), ABCG2 (i.e., BCRP or MXR), and ABCC1 (i.e., MRP1) transporters (Fletcher et al., 2016), which significantly decrease the intracellular levels of certain anticancer drugs by extrusion from cancer cells, thereby decreasing or even abolishing their efficacy (Wu and Fu, 2018). The ABCB1 transporter was discovered in 1976 and is the most studied ABC transporter. It is constitutively expressed in the blood-brain barrier, kidneys and liver, to protect normal cells from xenobiotic compounds. When overexpressed in cancer cells, ABCB1 produces an MDR phenotype to various chemotherapeutic drugs, including paclitaxel, doxorubicin, and tyrosine kinase inhibitors (TKIs) such as GSK-1070916 and TAK243 (Wu et al., 2020c; Wu et al., 2022). Over the past 4 decades, researchers have developed three generations of ABCB1 inhibitors, but their reversal effect was limited by their toxicity and/or low efficacy *in vivo*. Recently, it was reported that some TKIs, such as GS-9973, and sitravatinib, inhibit the efflux function of ABCB1, thereby reversing, reversing drug resistance (Yang et al., 2020; Narayanan et al., 2021). Similarly, ABCG2 is also a major mediator of MDR in cancer cells. ABCG2 mediates the efflux of the chemotherapeutic drugs, mitoxantrone, topotecan, irinotecan, and various tyrosine kinase inhibitors (TKIs), such as tivantinib and pevonedistat (Wu et al., 2020a; Wei et al., 2020). In contrast to ABCB1, the development of ABCG2 inhibitors has been significantly slower. In addition to the commonly used ABCG2 inhibitors, Ko143 and fumitremorgin C, recent studies showed that the TKIs, poziotinib, and CC-671, inhibit the efflux function of ABCG2 (Wu et al., 2020b; Zhang et al., 2020b). However, none of these inhibitors have been evaluated in clinical trials.

It is well known that unregulated cell division can lead to the development and progression of cancer (Sherr, 1996). Cyclin-dependent kinases (CDKs) are a family of serine/threonine protein kinases that regulate cell cycle and gene transcription (Manning et al., 2002). The CDK family can be divided into two major groups: 1) those that regulate cell cycle progression and interact with multiple cyclins, i.e., CDK1, CDK2, CDK4 and CDK6 and 2) those that regulate transcription and interact only with a single cyclin, i.e., CDK7, CDK8, CDK9, CDK11, CDK12, and CDK13 (Schmitz and Kracht, 2016; Wood and Endicott, 2018; Liang et al., 2020). The cell cycle can be divided into four successive phases: G1 (pre-DNA synthesis), S (DNA synthesis phase), G2 (late DNA synthesis), and M (mitotic phase) (Xu et al., 2018). The cyclin D-CDK4/6-RB-p16^INK4A^ pathway is a key regulatory pathway in the transition from G1 to S phase (Basso and Doll, 2006; Asghar et al., 2015). The dysregulation of this pathway facilitates cancer cell cycle progression and proliferation (Shapiro, 2006; Burkhart and Sage, 2008; Knudsen and Knudsen, 2008). CDK4/6 is a key regulator of the transition from G1 to S phase (Yuan et al., 2021) and the inhibition of CDK4/6 blocks progression of the cell cycle from the G1 to S phase in cancer cells (Anders et al., 2011; O’Leary et al., 2016; Kato et al., 2021). The CDK 4/6 inhibitor, palbociclib, in combination with other drugs, has been approved by the FDA to treat metastasized breast cancer that is estrogen receptor (ER) positive and human epidermal growth factor receptor 2 (HER2) negative (Ciruelos et al., 2020). The inhibition of CDK4/6 by palbociclib inhibits the phosphorylation of the retinoblastoma protein (RB), thereby blocking the progression of the cell cycle from the G1 to S phase, which inhibits cancer cell proliferation (Weinberg, 1995; Spring et al., 2020). A recent clinical trial reported that in patients with previously untreated ER-positive, HER2-negative advanced breast cancer, the combination of palbociclib with letrozole produced a longer progression-free survival than letrozole alone (Finn et al., 2016). Another trial reported that palbociclib, in combination with fulvestrant, improved the overall survival compared to fulvestrant alone, in ER-positive, HER2-negative advanced breast cancer patients (Turner et al., 2018). Although palbociclib and endocrine therapy is an effective treatment for ER-positive and HER2-negative metastatic breast cancer, the addition of palbociclib to standard endocrine therapy did not significantly improve treatment outcomes, compared to endocrine therapy alone (Finn et al., 2015; Cristofanilli et al., 2016; Harbeck et al., 2016; Malorni et al., 2018; Rugo et al., 2018; Johnston et al., 2019; Gnant et al., 2021; Mayer et al., 2021).

Previously it has been reported that in *ABCB1* and *ABCG2*-transfected Madin-Darby canine kidney II (MDCKII) cell lines, palbociclib was a substrate for the ABCB1 and ABCG2 transporters (Parrish et al., 2015). Similarly, the penetration of palbociclib across the blood-brain barrier of *ABCB1* and *ABCG2* gene knockout mice was significantly decreased by ABCB1 and ABCG2 transporters, suggesting that palbociclib was a substrate for ABCB1 and ABCG2 transporters (De Gooijer et al., 2015). In contrast, studies by Gao et al. (2017) and Sorf et al. (2020) suggested that palbociclib may be an inhibitor of the ABCB1 transporter. Therefore, it is crucial to determine if palbociclib is a substrate or an inhibitor because if it is a substrate of the ABCB1 transporter, cancer cells that overexpress ABCB1 transporter will have lower intracellular levels of palbociclib, which could decrease or abrogate its efficacy. Therefore, in this study, we conducted *in vitro* experiments and to determine if palbociclib is an inhibitor or a substrate of the ABCB1 or ABCG2 transporter.

## Materials and Methods

### Reagents

Colchicine, doxorubicin (adriamycin), mitoxantrone, and verapamil were purchased from Sigma Aldrich Trading Co. (Shanghai, China). All of the drugs were dissolved in 100% DMSO to produce a 10 mM stock solution.

### Cell Lines and Cell Culture

The human epidermoid carcinoma cell line, KB-3-1, was used as the parental cell line, and its colchicine–selected subline KB-C2, which overexpresses the ABCB1 transporter (Fojo et al., 1985), was used as a drug-resistant cell line. The human colon cancer cell line, SW620, was used as the parental cell line, and the doxorubicin-selected subline, SW620/Ad300, which overexpresses the ABCB1 transporter (Lai et al., 1991a; Lai et al., 1991b), was used as another drug-resistant cell line. We also used SW620 parental and SW620/Ad300 drug-resistant cell sublines, where the gene for the *ABCB1* transporter was knocked out using the CRISPR/Cas9 system (Lei et al., 2021). The human large cell lung cancer cell line, NCI-H460, was used as the parental cell line and its mitoxantrone-selected subline, NCI-H460/MX20, which overexpresses the ABCG2 transporter (Henrich et al., 2007), was used as another drug-resistant cell line. The drug resistance of the KB-C2, SW620/Ad300 and NCI-H460/MX20 cells was maintained in media containing 2 µg/ml of colchicine, 300 ng/ml of doxorubicin and 20 nM of mitoxantrone, respectively. The HEK293/pcDNA3.1, HEK293/ABCB1, and HEK293/ABCG2-WT cell lines were transfected with the empty vector pcDNA3.1, the pcDNA3.1 vector containing a full-length gene coding for the ABCB1 transporter and the pcDNA3.1 vector containing a full-length gene coding for the wild-type (WT) ABCG2, respectively (Robey et al., 2008; Mi et al., 2010). All gene transfected cell lines were maintained in a medium containing 2 mg/ml of G418, an aminoglycoside derivative used for the selection of eukaryotic expression vectors containing the related resistance genes (Robey et al., 2003). All the MDR cell lines were cultured in complete medium, containing 10% fetal bovine serum and 1% of penicillin/streptomycin and incubated at 37°C in an incubator with 5% CO_2_. Prior to the experiment, all the cells were cultured in drug-free complete medium for at least 2 weeks.

### Cytotoxicity Assay

The efficacy of palbociclib was determined using the MTT assay, which assesses cytotoxicity, as previously described (Zhang et al., 2016). Briefly, the cells were seeded in 96-well plates and incubated with different concentrations of palbociclib (0.03–100 μM) in the presence or absence of 3 μM of verapamil. The 96-well plates were incubated for 72 h. On the last day, MTT solution (5 mg/ml) was added to each well and the cells were incubated for 4 h at 37°C. At the end of the incubation, the supernatant was discarded and 100 μL of DMSO was added to each well to dissolve the purple formazan crystals. Subsequently, the absorbance at 570 nm was read using a AccuSkan^™^ GO UV/Vis Microplate Spectrophotometer (Thermo Fisher Scientific Inc., Waltham, MA, United States).

### ABCB1 ATPase Assay

An ATPase assay of kit (TEBU-BIO nv, Boechout, Belgium) was used to determine the effect of palbociclib on basal ABCB1 ATPase activity, as previously described (De Bruijn et al., 1986). Briefly, High Five insect cell membrane vesicles, containing the ABCB1 transporter, were incubated in microcentrifuge tubes at 37°C for 5 min in the supplied assay buffer, in the presence or absence of the phosphorotyrosol phosphatase inhibitor, Na_3_VO_4_. Subsequently, the cells were incubated with vehicle (DMSO) or 0.1–20 μM of palbociclib for 3 min at 37°C. Subsequently, 5 mM of Mg^2+^ATP was added to activate ATP hydrolysis, and 20 min later, the reaction was terminated by adding 0.1 ml of a 5% sodium dodecyl sulfate solution. The inorganic phosphate produced by the activation of the ATPase was measured as previously described (Ambudkar, 1998; Shi et al., 2007; Shi et al., 2011). In a separate experiment, High Five insect cell membrane vesicles were incubated with palbociclib (0.1–20 μM) and 3 μM of tepotinib, an inhibitor of the ABCB1 transporter, and ATPase activity was determined as above mentioned.

### [^3^H]-Paclitaxel Accumulation Assay

The intracellular accumulation of [^3^H]-paclitaxel in the KB-3-1 parental and KB-C2 resistant cell lines was used to determine the effect of palbociclib on ABCB1-mediated drug efflux activity, following the same protocol as previously described (Wu et al., 2020c). The samples were assayed for radioactivity using a Packard TRI-CARB 1900 CA liquid scintillation analyzer (Packard Instrument, Downers Grove, IL).

### Western Blot Analysis

Drug-resistant KB-C2 and SW620/Ad300 cells were incubated with 0.3–5 μM of palbociclib for up to 72 h at 37°C and the corresponding parental cells KB-3-1 and SW620 were used as negative control. The cell lysates were prepared by adding RIPA lysis buffer to the cells, then centrifuged at 12,000 g, 4°C for 20 min to collect the supernatant. The protein concentration in the lysates was determined using the bicinchoninic acid assay, as previously described (Wang et al., 2020a). Protein samples were subjected to SDS-PAGE, transferred to PVDF membrane and incubated with the primary antibodies (anti-ABCB1 antibody and anti-GAPDH antibody) overnight at 4°C after being blocked with 5% skim milk powder for 2 h at room temperature. The next day, after washing the PVDF membranes with TBST buffer, the PVDF membranes were incubated with horseradish peroxidase-labeled secondary antibodies (anti-mouse antibody, 1:1,000 dilution) for 2 h at room temperature and the immunoreactive bands were visualized by chemiluminescence by using an enhanced chemiluminescence detection system (Amersham, NJ, United States). Finally, the results were quantified using ImageJ software, which was used to determine the relative density of the immunoreactive bands.

### Molecular Docking of Palbociclib With Human ABCB1 Models

The 3-D structure of palbociclib was established for docking simulations, using a human ABCB1 model, as previously described (Ji et al., 2018; Zhang et al., 2020a; Zhang et al., 2020b). The human ABCB1 protein model 6QEX (paclitaxel bound) was acquired from Protein Data Bank. The model was an inward-facing human ABCB1 with a resolution of 3.6 Å (6QEX). Docking grid (length: 30 Å) center coordinates were defined by setting the centroid with the amino acid residues that are suggested to interact with paclitaxel, an ABCB1 substrate. The receptor and ligand preparations and docking simulations were done using the default settings in Maestro v11.1 (Schrodinger, LLC, Cambridge, MA). The top-scoring pose, expressed as kcal/mol, was selected for further analysis and visualization.

### Statistical Analysis

All experiments were repeated at least three times and IC_50_ values were expressed as the mean ± standard deviation (SD) of at least three independent experiments. All data were expressed as mean ± SD and statistically analyzed using a one-way analysis of variance (ANOVA), followed by Tukey’s *post hoc* test. GraphPad Prism 8.1 software was used in to plot the graphs and analyze the data. The *a priori* significance levels were *p* < 0.05.

## Results

### The *in Vitro* Efficacy of Palbociclib was Significantly Decreased in Cells Overexpressing the ABCB1 Transporter

In order to ascertain if palbociclib is a substrate for the ABCB1 transporter, we determined the efficacy of palbociclib in the ABCB1 overexpressing cell lines, KB-C2, SW620/Ad300, and HEK293/ABCB1, using the MTT assay.

**FIGURE 1:**
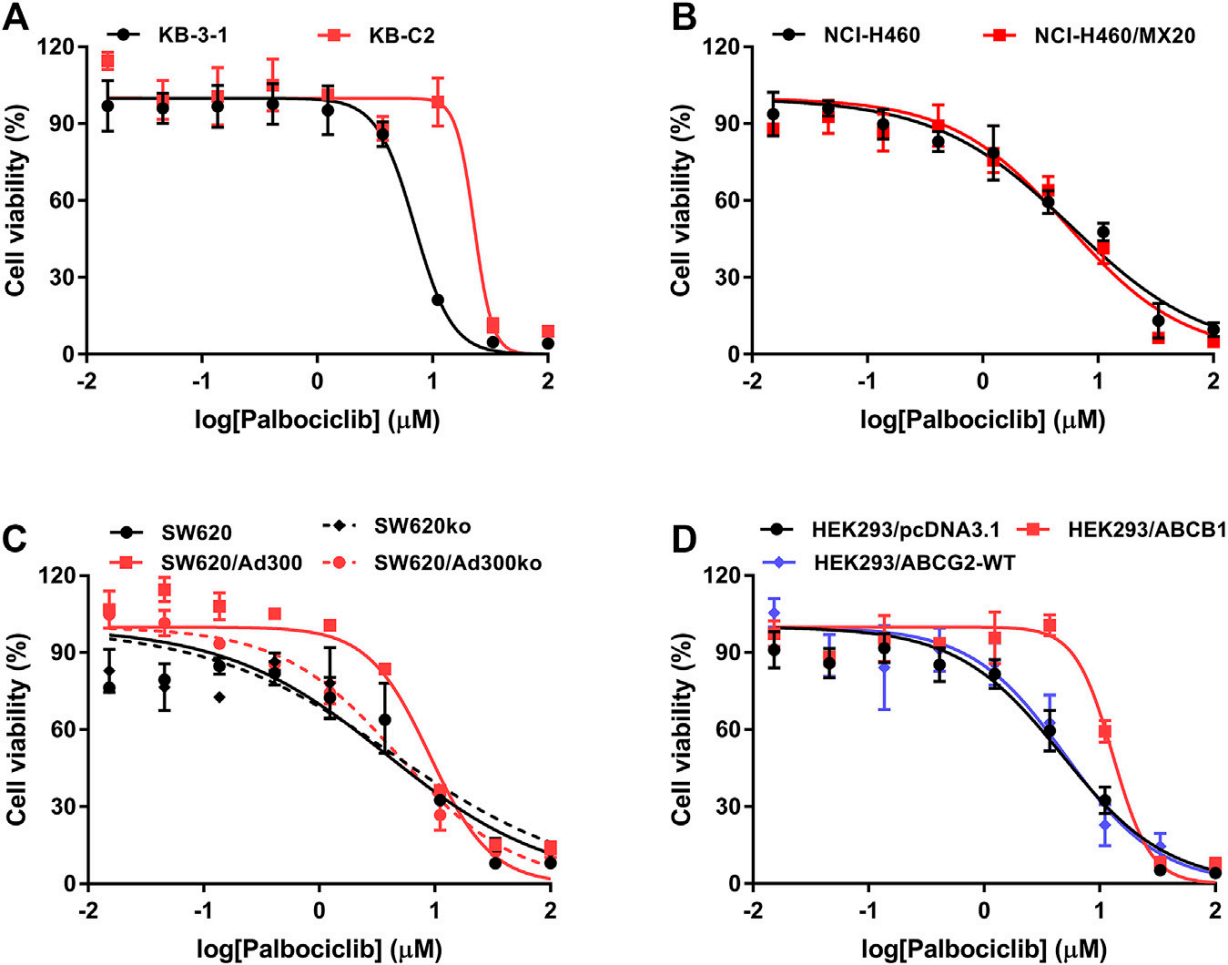
Cytotoxicity of palbociclib in parental and MDR cell lines. The efficacy of palbociclib in **(A)** KB-3-1 and KB-C2 cells, **(B)** NCI-H460 and NCI-H460/MX20 cells, **(C)** SW620, SW620/Ad300, SW620-ABCB1ko, and SW620/Ad300-ABCB1ko cells, and **(D)** HEK293/pcDNA3.1, HEK293/ABCB1, and HEK293/ABCG2-WT. All data are expressed as the mean ± SD of three independent experiments.

As shown in Figure 1, we determined the cytotoxicity of palbociclib in ABCB1-overexpressing KB-C2 (IC_50_ = 22.573 μM), SW620/Ad300 (IC_50_ = 9.045 μM), and HEK293/ABCB1 (IC_50_ = 13.855 μM) cells and their parental cell lines, KB-3-1 (IC_50_ = 5.014 μM), SW620 (IC_50_ = 3.921 μM), and HEK293/pcDNA3.1 (IC_50_ = 4.071 μM), respectively.

As shown in Table 1 and Figures 1 A, C, D, compared to the corresponding parental KB-3-1 and SW620 cells, there was a significant difference in the resistance of KB-C2 and SW620/Ad300 cells to palbociclib and the resistance-fold (RF) values were 4.50- and 2.31-fold, respectively. Similarly, compared to HEK293/pcDNA3.1 cells, there was a significant difference in the resistance of HEK293/ABCB1 cells to palbociclib and the RF value was 3.40-fold. These results indicated that overexpression of the ABCB1 transporter decreased the efficacy of palbociclib in drug-resistant cells. SW620 cells express a low endogenous level of the ABCB1 transporter and thus to eliminate the effect of the ABCB1 transporters on palbociclib level, we used the cells that did not contain the gene for the ABCB1 transporter, SW620-ABCB1ko and SW620/Ad300-ABCB1ko cells. There was no significant difference in the RF value for palbociclib between the non-modified (RF = 1) and *ABCB1* knockout SW620 cells (RF = 1.16). In contrast, the efficacy of palbociclib was increased in SW620/Ad300 cells that did not have the gene for the ABCB1 transporter (RF = 1.23), compared to the non-modified SW620/Ad300 cells (RF = 2.31). These results indicated that knocking out the *ABCB1* transporter gene increased the efficacy of palbociclib in the drug-resistant SW620/Ad300 cells to a magnitude similar to that of the parental SW620 cells. Overall, the above results indicated that the efficacy of palbociclib was significantly decreased in drug-resistant cells overexpressing the ABCB1 transporter.

**TABLE 1:** The cytotoxicity of palbociclib in parental and MDR cells.

Treatment	Overexpressed transporter	IC_50_ value ± SD^a^ (μM, resistance fold^b^)	
		Palbociclib	Palbociclib + verapamil 3 μM
KB-3-1	-	5.014 ± 1.407 (1.00)	5.745 ± 1.039 (1.15)
KB-C2	ABCB1	22.573 ± 4.424 (4.50)*	6.071 ± 0.293 (1.21)
SW620	-	3.921 ± 0.412 (1.00)	5.470 ± 0.739 (1.40)
SW620/Ad300	ABCB1	9.045 ± 0.297 (2.31)*	4.563 ± 0.766 (1.16)
SW620 ABCB1 ko	-	4.559 ± 0.836 (1.16)	6.812 ± 1.446 (1.74)
SW620/Ad300 ABCB1 ko	-	4.826 ± 0.23 (1.23)	5.952 ± 1.221 (1.52)
NCI-H460	-	5.598 ± 0.258 (1.00)	-
NCI-H460/MX20	ABCG2	5.258 ± 0.712 (0.94)	-
HEK293/pcDNA3.1	-	4.071 ± 0.738 (1.00)	4.948 ± 0.512 (1.22)
HEK293/ABCB1	ABCB1	13.855 ± 6.964 (3.40)*	5.371 ± 1.054 (1.32)
HEK293/ABCG2-WT	ABCG2	4.644 ± 0.306 (1.14)	-

a IC_50_ values represent the mean ± SD of at least three independent experiments.

b RF: Resistance-fold was calculated by dividing the IC_50_ values of the ABCB1 or ABCG2 substrate in the presence or absence of verapamil by the IC_50_ value for the parental cells in the absence of the verapamil.

*p < 0.05 versus the control group in the absence of the inhibitor.

### The Efficacy of Palbociclib is Not Significantly Altered in Cells Overexpressing the ABCG2 Transporter

In order to ascertain if palbociclib was a substrate for the ABCG2 transporter, we determined the efficacy of palbociclib in the ABCG2 overexpressing cell lines, NCI-H460/MX20 and HEK293/ABCG2-WT. Palbociclib was cytotoxic in NCI-H460/MX20 (IC_50_ = 5.258 μM) and HEK293/ABCG2-WT (IC_50_ = 4.644 μM) cells overexpressing the ABCG2 transporter and their parental cell lines, NCI-H460 (IC_50_ = 5.598 μM), and HEK293/pcDNA3.1 (IC_50_ = 4.071 μM), respectively.

As shown in Table 1 and Figures 1 B, D, compared to the corresponding parental NCI-H460 cells, there was no significant difference in the resistance of NCI-H460/MX20 cells to palbociclib (RF = 0.94). Similarly, compared to the corresponding empty vector control HEK293/pcDNA3.1 cells, there was no significant difference in the resistance of HEK293/ABCG2-WT cells to palbociclib (RF = 1.14). These results suggest that the overexpression of the ABCG2 transporter does not decrease the efficacy of palbociclib in drug-resistant cells.

### The ABCB1 Inhibitor, Verapamil, Restores the Efficacy of Palbociclib in Cells Overexpressing the ABCB1 Transporter

As shown in Table 1, in the presence of 3 μM of verapamil, an inhibitor of the ABCB1 transporter (Muller et al., 1994), the resistance–fold (RF) value of palbociclib in KB-C2 and SW620/Ad300 cancer cells was significantly decreased the from 4.50- to 1.21-fold and 2.31- to 1.16-fold, respectively. Similarly, in HEK293 cells that overexpress the ABCB1 gene (HEK293/*ABCB1*), palbociclib resistance was significantly decreased by 3 μM of verapamil (from 3.40- to 1.32-fold; Table 1). These results indicated that verapamil increased the efficacy of palbociclib in the resistant cancer cells to a magnitude similar to that of the parental cancer cells. In contrast, verapamil did not significantly alter the efficacy of palbociclib in the parental cell lines, KB-3-1, SW620, and HEK293/pcDNA3.1 cells, which do not overexpress the ABCB1 transporter. Thus, the resistance of KB-C2, SW620/Ad300 and HEK293/ABCB1 cells to palbociclib was due to the overexpression of the ABCB1 transporter. Finally, the IC_50_ values of palbociclib in the presence of verapamil for the knockout cell lines, SW620 *ABCB1* knockout and SW620/Ad300 *ABCB1* knockout, were not significantly different from the IC_50_ values of palbociclib alone, further indicating that SW620/Ad300 cells to palbociclib was due to the overexpression of the ABCB1 transporter.

### Palbociclib Stimulates the Basal Activity of the ABCB1 Transporter ATPase

**FIGURE 2:**
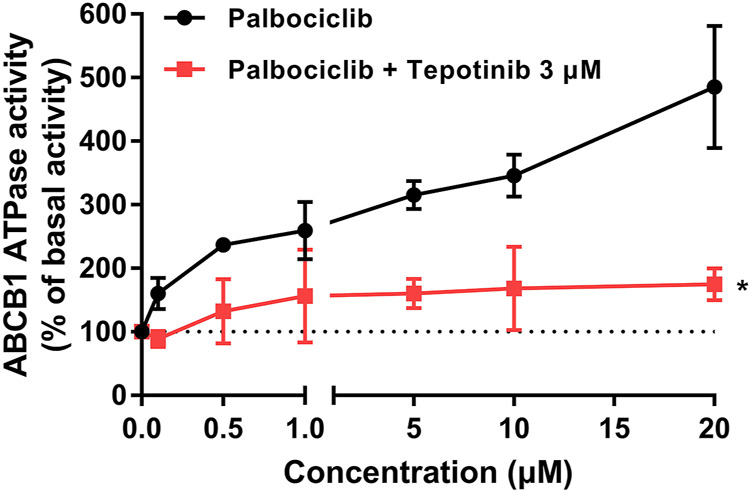
The effect of palbociclib on ABCB1 ATPase activity in the absence or presence of the ABCB1 ATPase inhibitor, tepotinib. The black line represents the basal ABCB1 ATPase activity after incubation with vehicle (0 μM) or palbociclib (0.1–20 μM). The red line represents the basal ABCB1 ATPase activity after incubation with vehicle (0 μM), palbociclib (0.1–20 μM) and tepotinib (3 μM). All data are expressed as mean ± SD from three independent experiments. *p < 0.05 versus the palbociclib single treatment.

Previously, it has been reported that ABCB1 substrates increase the hydrolysis of ATP, which is coupled to the efflux function of the ABCB1 transporter (Dastvan et al., 2019; Szöllősi et al., 2020). Therefore, we determined the effect of palbociclib on the basal activity of the ABCB1 ATPase in Insect High Five cell membranes. As shown in Figure 2, palbociclib produced a concentration-dependent (0.1–20 μM) increase in the basal activity of ABCB1 ATPase. Palbociclib produced a maximal increase of 4.85-fold in the basal level of ATPase activity, compared to cells incubated with the vehicle (Figure 2). At 4.73 μM, palbociclib produced a 50% increase in the basal ATPase activity.

To further support our hypothesis that palbociclib is a substrate of ABCB1, we determined the effect of tepotinib, an inhibitor of ABCB1 ATPase (Wu et al., 2019), on the palbociclib-induced increase in Insect High Five ABCB1 ATPase activity. The results indicated that tepotinib significantly inhibited the palbociclib-induced increase in ABCB1 ATPase activity. Overall, these results suggest that palbociclib is a substrate for the ABCB1 transporter.

### Palbociclib Increases the Accumulation of the ABCB1 Transporter Substrate, [^3^H]-paclitaxel

**FIGURE 3:**
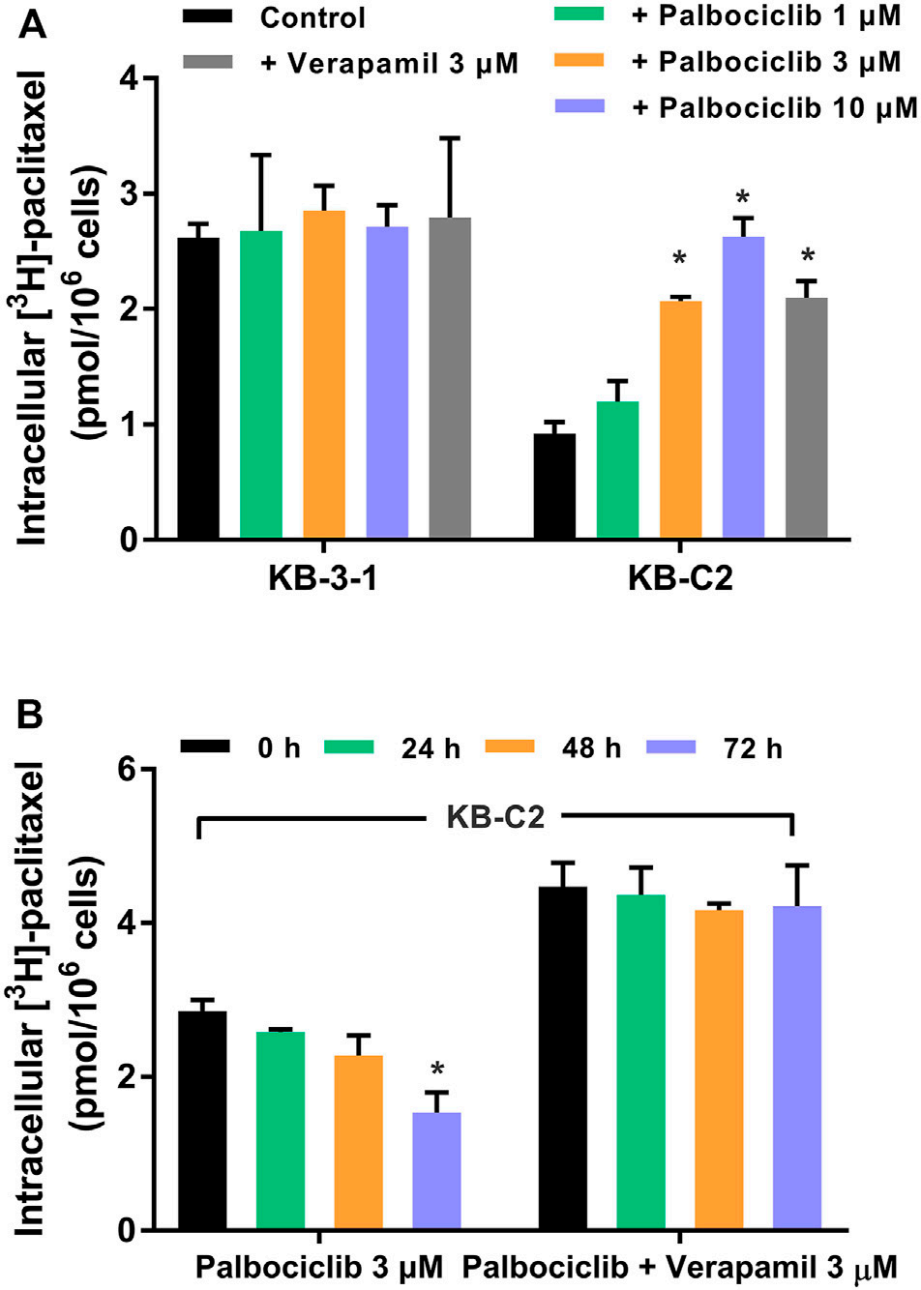
The effect of palbociclib on the intracellular accumulation of [^3^H]-paclitaxel in KB-3-1 and KB-C2 cells. **(A)** KB-3-1 and KB-C2 cells were incubated with either vehicle (control group), 3 μM of verapamil or 1, 3, or 10 μM of palbociclib for 2 h, followed by incubation with [^3^H]-paclitaxel for 2 h. **(B)** Left side bar graphs: KB-C2 cells were incubated with 3 μM of palbociclib and [^3^H]-paclitaxel for 0, 24, 48, or 72 h and the intracellular accumulation of [^3^H]-paclitaxel was determined using liquid scintography. Right side bar graphs: KB-C2 cells were incubated with 3 μM of palbociclib, 3 μM of verapamil (an inhibitor of the ABCB1 transporter), and [^3^H]-paclitaxel for 24, 48, 72 h) and the intracellular accumulation of [^3^H]-paclitaxel was determined using liquid scintography. All data are expressed as mean ± SD based on three independent experiments. *p < 0.05 compared to the corresponding control group.

To further validate our hypothesis that palbociclib is a substrate for the ABCB1 transporter, we determined the effect of palbociclib on the intracellular levels of further assess the interaction between palbociclib and [^3^H]-paclitaxel, a substrate of ABCB1 transporter (Gao et al., 2001; Taub et al., 2005). As previously reported (Cui et al., 2019a; Cui et al., 2019b; Li et al., 2020), our results indicated that the accumulation of [^3^H]-paclitaxel in the parental cancer cell line, KB-3-1, which does not overexpresses the ABCB1 transporter, was significantly greater than that of KB-C2 drug-resistant cancer cell line, due to an increase in the extrusion of [^3^H]-paclitaxel from the cancer cells (Figure 3A). The incubation of KB-3-1 cells for 2 h with either palbociclib (1, 3, or 10 μM) or verapamil (3 μM) did not significantly alter the intracellular accumulation of [^3^H]-paclitaxel (Figure 3A), as these cells do not overexpress the ABCB1 transporter. In contrast, in KB-C2 cells, 3 and 10 μM of palbociclib, compared to vehicle, significantly increased the accumulation of [^3^H]-paclitaxel (Figure 3A) Similarly, the incubation of KB-C2 cells with 3 μM of verapamil significantly increased the intracellular accumulation of paclitaxel compared to cells incubated with vehicle (Figure 3A). Palbociclib, at 1 μM, produced a lower inhibition of the ABCB1 transporter, compared to 3 μM of verapamil (Figure 3A). However, 3 and 10 μM of palbociclib increased the accumulation of [^3^H]-paclitaxel in KB-C2 cells to a level similar to that of the parental KB-3-1 cells (Figure 3A).

As shown in Figure 3B, the incubation of KB-C2 cells with 3 μM of palbociclib for 24 or 48 h did not significantly alter the accumulation of intracellular [^3^H]-paclitaxel compared to 0 h. However, the incubation of KB-C2 cells with 3 μM of palbociclib for 72 h significantly decreased the accumulation of [^3^H]-paclitaxel compared to 0 h (Figure 3B). This effect could have been due to a palbociclib-induced increase in the expression level of the ABCB1 transporter (see *Palbociclib Upregulates the Expression of the ABCB1 Transporter Protein* below).

The incubation of KB-C2 cells with 3 μM of palbociclib and 3 μM of verapamil for 24, 48, or 72 h significantly increased the accumulation of [^3^H]-paclitaxel, compared to KB-C2 cells incubated with only palbociclib (Figure 3B). These results suggest that the overexpression of the ABCB1 transporter was the primary cause for the decreased accumulation of [^3^H]-paclitaxel in the KB-C2 cells.

### Palbociclib Upregulates the Expression of the ABCB1 Transporter Protein

**FIGURE 4:**
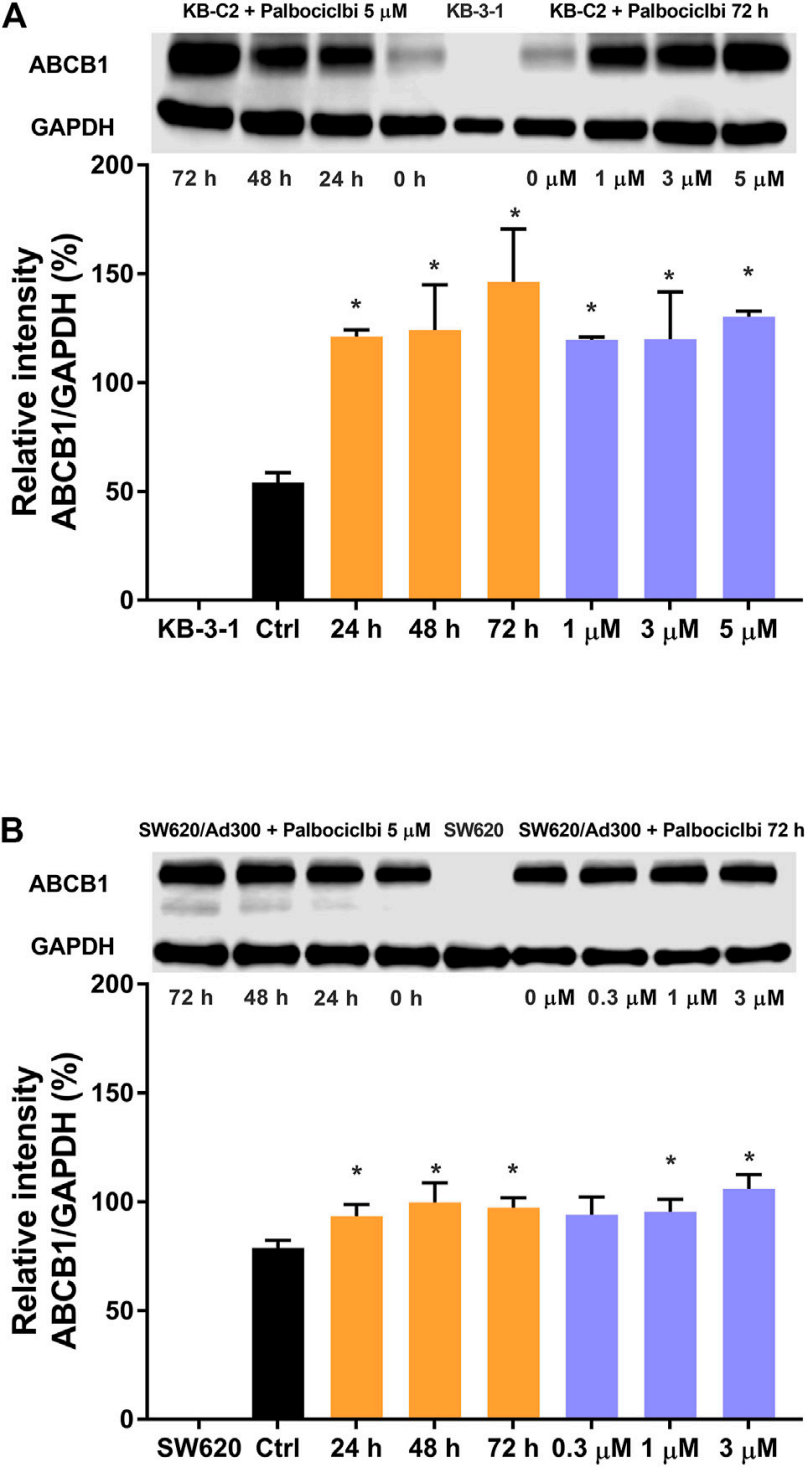
The effect of palbociclib on the expression level of the ABCB1 protein in cancer cell lines. **(A)** Left panel: The effect of the incubation of vehicle (0 μM) or 5 μM of palbociclib for 24, 48, or 72 h on the expression level of the ABCB1 protein in KB-C2 cells. **(A)** Right panel: The effect of the incubation of vehicle (0 μM) or 1, 3, or 5 μM of palbociclib for 72 h on the expression level of the ABCG2 protein in KB-C2 cells **(B)** Left panel: The effect of the incubation of vehicle (0 μM) or 3 μM of palbociclib for 24, 48, or 72 h on the expression level of the ABCB1 protein in SW620/Ad300 cells. **(B)** Right panel: The effect of the incubation of vehicle (0 μM) or 0.3, 1, or 3 μM of palbociclib for 72 h on the expression level of the ABCG2 protein in SW620/Ad300 cells. All data are expressed as the mean ± SD based on three independent experiments. *p < 0.05 compared to the corresponding control group.

In the above experiments, one of the results indicated that 3 μM of palbociclib significantly decreased the intracellular levels of [^3^H]-paclitaxel in the drug resistant KB-C2 cancer cells. It is possible that this could have been due to a palbociclib-induced increase in the expression level of the ABCB1 transporter. Therefore, we determined the *in vitro* effect of palbociclib on the expression of the ABCB1 protein in KB-C2 cells using the western blotting assay. The incubation of KB-C2 cells with 5 μM of palbociclib for 24, 48, or 72 h, significantly increased the levels of the ABCB1 protein, compared to KB-C2 cells incubated with vehicle (control, 0 μM; Figure 4A, left panel). In contrast, there was no band in the western blot assay for the parental KB-3-1 cells, a finding consistent with the fact that these cells do not overexpress the ABCB1 transporter. The incubation of KB-C2 cells with 1, 3, or 5 μM of palbociclib also significantly increased the expression of the ABCB1 protein compared to KB-C2 cells incubated with vehicle (control, 0 μM; Figure 4B, right panel).

We also determined the effect of palbociclib on the expression of the ABCB1 protein in SW620/Ad300 cancer cells, which also overexpress the ABCB1 transporter. Similar to the results for KB-C2 cells, the incubation of SW620/Ad300 cells with 3 μM of palbociclib significantly increased the expression of the ABCB1 protein compared to cells incubated with vehicle (control, 0 μM; Figure 4B left panel). However, no band for the ABCB1 protein was obtained for the SW620 parental cancer cells, as they did not overexpress the ABCB1 transporter. The incubation of SW620/Ad300 cells with 1 or 3 μM of palbociclib for 72 h also significantly increased the expression of the ABCB1 protein (Figure 4B, right panel).

### Docking Simulation of Palbociclib in the Drug-Binding Area of Human ABCB1

**FIGURE 5:**
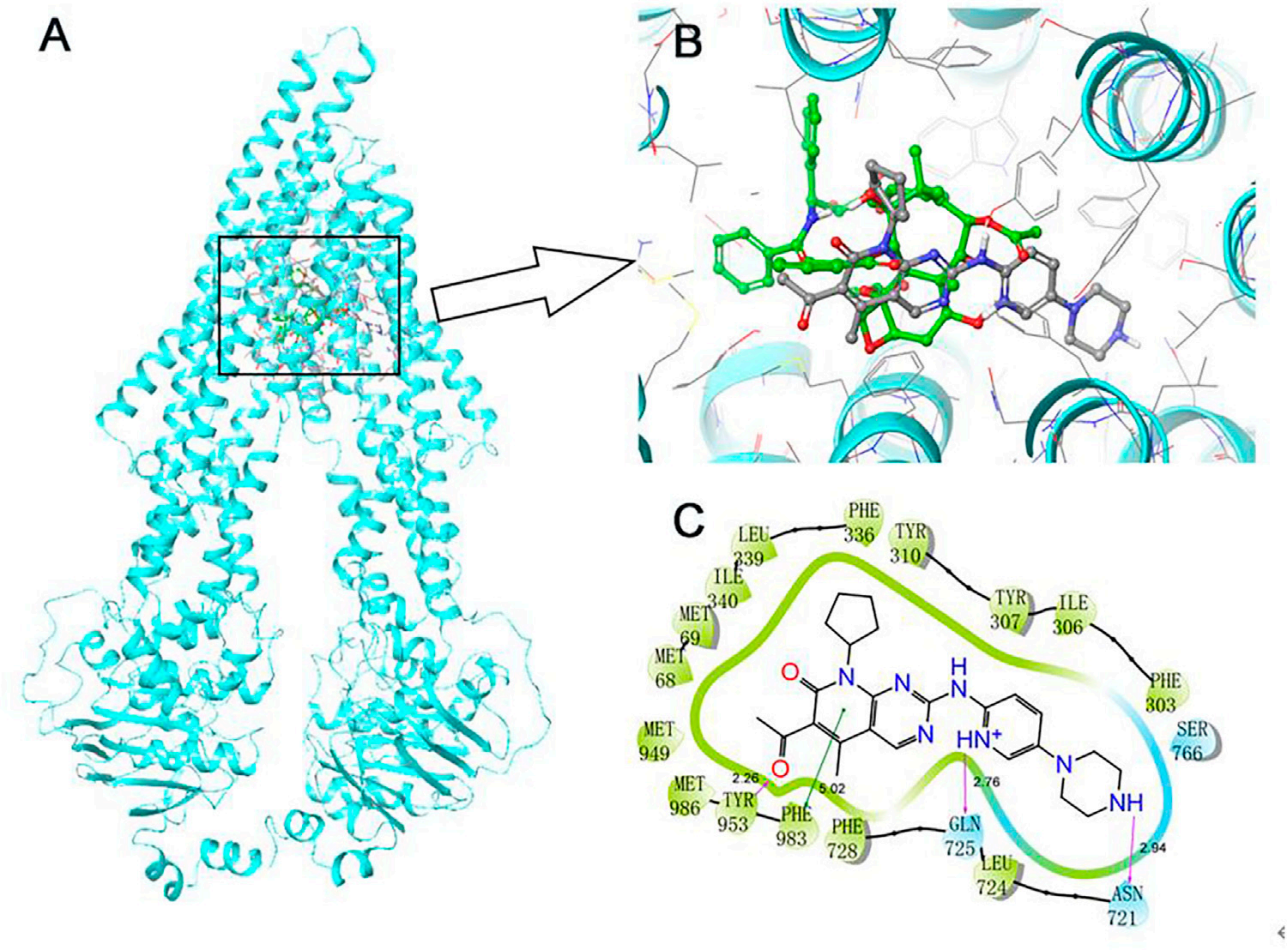
Induce-fitted docking predicted binding poses for palbociclib in the human ABCB1 model. **(A)** A ribbon diagram (cyan) of homology model of human ABCB1 and the location of palbociclib and paclitaxel are shown within the ABCB1 internal cavity. **(B)** The detailed interactions of palbociclib (green) and paclitaxel (grey) are depicted as a ball and stick model with the ABCB1 residues. **(C)** A 2D schematic diagram of ligand–receptor interaction between palbociclib and the human ABCB1 model. Amino acids within 3 Å are depicted in colored bubbles, polar residues are depicted in light blue and hydrophobic residues are depicted in green. Purple arrows denote H-bonds, and the green lines indicate π-π interactions. The relevant distances are in Å.

A docking simulation of the paclitaxel binding site (6QEX) of ABCB1 protein was performed. A docking simulation of palbociclib with paclitaxel-bound ABCB1 protein model (6QEX) was performed. The results showed that palbociclib has multiple interactions with the substrate binding site for the ABCB1 protein and an affinity score of -9.616 kcal/mol (Figure 5). Palbociclib was positioned and stabilized in the hydrophobic cavity formed by Met68, Met69, Phe303, Ile306, Tyr307, Tyr310, Phe336, Leu339, Ile340, Asn721, Leu724, Gln725, Phe728, Ser766, Met949, Tyr953, Phe983, and Met986. Therefore, hydrophobic interactions played an important role in the binding of palbociclib with ABCB1 protein. Furthermore, the formation of hydrogen bonds with Asn721, Gln725, and Tyr953 contributed to the stability of the carbonyl group and the amide group of palbociclib and the pyridine group of palbociclib was stabilized by the formation of a π-π bond with the phenol ring of Phe983 of ABCB1.

## Discussion

Numerous studies have shown that the overexpression of the ABC transporters, ABCB1 and ABCG2, in certain types of solid tumors and leukemia, produce acquired drug resistance and attenuate, as well as abrogate, the efficacy of anticancer drugs with different chemical structures and mechanisms of action (Mohammad et al., 2018; Boyer et al., 2019; Wu et al., 2020c; Yang et al., 2021). It has been reported that palbociclib, a CDK4/6 inhibitor, may be a substrate of the ABCB1 and ABCG2 transporters (De Gooijer et al., 2015; Parrish et al., 2015) and an ABCB1 chemosensitizer (Fojo et al., 1985; Gao et al., 2017; Sorf et al., 2020). However, the exact interaction palbociclib with the ABCB1 and ABCG2 transporters remained to be determined, as this depends on the concentration range of the drug, the methodology and the activity of the transporters in the various cell types (Yang et al., 2021). Thus, given that the overexpression of MDR-associated ABC transporters is still an issue in cancer treatment, identifying drugs that can be pumped out from cancer cells could help to produce the maximal efficacy of cancer therapy, thereby potentially improving the patients’ quality of life. Therefore, in this study, we conducted experiments to determine the interaction of palbociclib with ABCB1 and ABCG2 transporters.

One of the main findings of this study was that the efficacy of palbociclib was significantly lower, as indicated by the higher IC_50_ values, in the drug-selected cancer cell lines, KB-C2 and SW620/Ad300, which overexpress the ABCB1 transporter, compared to the drug-sensitive parental cancer cell lines, KB-3-1 and SW620, which do not overexpress the ABCB1 transporter. Furthermore, the incubation of the ABCB1 overexpressing cell lines with 3 μM of verapamil, an inhibitor of the ABCB1 transporter, increased the efficacy of palbociclib similar to that of their respective parental cell lines. Similarly, the efficacy of palbociclib was decreased in HEK293 cells transfected with the *ABCB1* gene, where drug resistance was mediated only by the overexpression of the ABCB1 transporter. This finding was further confirmed by the fact that the knockout of the *ABCB1* gene reversed the resistance to palbociclib (i.e., its efficacy was restored) produced by the overexpression of the ABCB1 transporter. However, the efficacy of palbociclib was not significantly altered in either the drug-selected or the gene-transfected cells overexpressing the ABCG2 transporter, as indicated by the similar IC_50_ values for palbociclib in the drug-resistant and drug-sensitive cells. Overall, these results indicated that the overexpression of ABCB1, not the ABCG2 transporter, significantly decreased the efficacy of palbociclib. Subsequently, we conducted experiments to determine the mechanism of resistance to palbociclib in cells overexpressing the ABCB1 transporter.

Cancer cell resistance to anticancer drugs can occur due to an increase in the expression of the certain ABC transporters (Chen et al., 2021). Therefore, we used western blot assays to determine if palbociclib upregulates the expression level of the ABCB1 transporter. Our results indicated that the incubation of the drug resistant KB-C2 and SW620/Ad300 cells, which overexpress the ABCB1 transporter, with 5 μM of palbociclib for 24, 48, or 72 h, significantly increased the expression of the ABCB1 protein compared to the control group. Furthermore, the incubation of KB-C2 and SW620/Ad300 cells with 1, 3, or 5 μM of palbociclib for 72 h, significantly increased the expression of the ABCB1 protein, compared to SW620/Ad300 cells without treatment. Overall, these results suggest that palbociclib resistance may be due to an increase in the level of the ABCB1 protein.

It has been reported that the hydrolysis of ATP is coupled to the drug efflux function of the ABCB1 transporter (Yang et al., 2020). Consequently, we conducted an ATPase assay to determine if palbociclib increases the vanadate-sensitive ATPase activity of the ABCB1 transporter. Our results indicated that palbociclib can increase the ABCB1 ATPase activity in a concentration-dependent manner. The maximum stimulation was 4.85-fold greater than the basal level of ABCB1 ATPase activity. This increase is congruent with other studies reporting that drugs that are ABCB1 substrates, such as GSK-1070916 (2.6-fold) (Wu et al., 2020c) and volasertib (3.0-fold) (Wu et al., 2015). Furthermore, the incubation of High Five Insect cell membrane expressing ABCB1 ATPase with 3 μM of tepotinib, an inhibitor of ABCB1 ATPase activity (Wu et al., 2019), decreased the palbociclib-induced increase in ATPase activity to that of membranes incubated with palbociclib, i.e., the basal activity. These results suggest that palbociclib is a substrate of the ABCB1 transporter.

To further characterize the interaction between palbociclib and ABCB1 transporter, the [^3^H]-paclitaxel accumulation assay was performed to evaluate the effect of palbociclib on ABCB1 efflux activity. Previously, it has been reported that [^3^H]-paclitaxel is a substrate for the ABCB1 transporter (Zhou, 2008). The incubation of KB-C2 cells, which overexpress the ABCB1 transporter, with 3 or 10 μM of palbociclib, for 2 h, significantly increased the intracellular level of [^3^H]-paclitaxel. Similarly, the intracellular accumulation of [^3^H]-paclitaxel was increased by 3 μM of verapamil, a known inhibitor of the ABCB1 transporter. In contrast, palbociclib did not significantly alter the intracellular accumulation of [^3^H]-paclitaxel in the corresponding parental KB-3-1 cells, which do not overexpress the ABCB1 transporter. Based on these results, we hypothesized that palbociclib may compete with [^3^H]-paclitaxel and thus, decrease the efflux of [^3^H]-paclitaxel for the KB-C2 cells that overexpress the ABCB1 transporter. However, this hypothesis remains to be further validated. Since the western blot results indicated that palbociclib upregulated the protein expression of ABCB1 transporter, it was necessary to determine if the drug resistant cells had a higher resistance phenotype towards ABCB1 substrate drugs after incubation with palbociclib. Therefore, we incubated KB-C2 cells for 72 h with 3 μM of palbociclib and measured the intracellular levels of [^3^H]-paclitaxel. Our results showed that the KB-C2 cells accumulated a significantly lower level of [^3^H]-paclitaxel, suggesting that palbociclib increased the efflux activity of the ABCB1 transporter. This finding is validated by our results indicating that the ABCB1 inhibitor, verapamil, which inhibits the efflux function of the ABCB1 transporter, increased the intracellular accumulation of [^3^H]-paclitaxel to that of cells incubated with vehicle. It should be noted that, while palbociclib upregulates ABCB1 expression, the accumulation assay was conducted by incubating the cells with palbociclib for 2 h. Therefore, palbociclib is unlikely to modulate the expression of the ABCB1 protein within the short period of time, thereby allowing us to determine the binding of the substrate to the ABCB1 transporter.

Computational molecular docking analysis is widely used to predict ligand-protein interactions, even though it does not depict the actual binding interaction of the ligand (i.e., drug) with the protein (Salmaso and Moro, 2018). Recently, docking simulation has become a reliable method in screening for drugs that may be modulators or substrates of the ABC transporters (Wu et al., 2020c; Yang et al., 2020). We used an *in silico* molecular docking analysis to simulate the interaction of palbociclib and the ABCB1 protein. We postulated that palbociclib significantly increased ABCB1 ATPase activity due to its interaction at the drug-substrate binding domain of ABCB1 and therefore, we used the ABCB1 protein (6QEX) in this study. The docking analysis indicated that the docking score of palbociclib for the ABCB1 protein was −9.616 kcal/mol, which is comparable to that of other known ABCB1 substrates, such as GSK-1070916 (−8.0 kcal/mol) (Wu et al., 2020c) and WYE-354 (−12.241 kcal/mol) (Wang et al., 2020b). Also, our results showed that palbociclib is positioned in the hydrophobic cavity of the transmembrane domain formed by the amino acid residues and stabilized by hydrogen bonding interactions with Asn721, Gln725, Tyr953, and π- π interaction formed by the phenol ring with Phe983 of ABCB1 protein.

## Conclusion

Overall, our *in vitro* results indicate that the anticancer efficacy of palbociclib is significantly decreased by the overexpression of the ABCB1 transporter. In addition, it is likely that palbociclib is a substrate for the ABCB1 transporter, as palbociclib increased the ATPase activity of the ABCB1 transporter and upregulated the expression of the ABCB1 protein. Furthermore, the cytotoxic efficacy of palbociclib in drug resistant cells was restored by an ABCB1 inhibitor or the deletion of the *ABCB1* gene.

## Data Availability

The original contributions presented in the study are included in the article/Supplementary Material, further inquiries can be directed to the corresponding authors.
